# Design of experiment (DOE) applied to artificial neural network architecture enables rapid bioprocess improvement

**DOI:** 10.1007/s00449-021-02529-3

**Published:** 2021-02-27

**Authors:** Daniel Rodriguez-Granrose, Amanda Jones, Hannah Loftus, Terry Tandeski, Will Heaton, Kevin T. Foley, Lara Silverman

**Affiliations:** 1 DiscGenics Inc, Salt Lake City, Utah USA; 2 Department of Biochemistry and Molecular Biology, University of Miami, Miami, FL USA; 3 Department of Neurosurgery, University of Tennessee Health Science Center, Memphis, TN USA; 4 Semmes-Murphey Clinic, Memphis, TN USA

**Keywords:** Bioprocess, Artificial neural network, Process modeling, MachineLearning, Design of experiments (DOE)

## Abstract

Modern bioprocess development employs statistically optimized design of experiments (DOE) and regression modeling to find optimal bioprocess set points. Using modeling software, such as JMP Pro, it is possible to leverage artificial neural networks (ANNs) to improve model accuracy beyond the capabilities of regression models. Herein, we bridge the gap between a DOE skill set and a machine learning skill set by demonstrating a novel use of DOE to systematically create and evaluate ANN architecture using JMP Pro software. Additionally, we run a mammalian cell culture process at historical, one factor at a time, standard least squares regression, and ANN-derived set points. This case study demonstrates the significant differences between one factor at a time bioprocess development, DOE bioprocess development and the relative power of linear regression versus an ANN-DOE hybrid modeling approach.

**Supplementary Information:**

The online version contains supplementary material available at 10.1007/s00449-021-02529-3.

## Introduction

Due to multivariate datasets, sophisticated data interpretation is increasingly attractive to bioprocess professionals [1–3]. Traditional regression techniques have limitations, including the shapes that they can use to model data, poor extrapolation properties, and sensitivity to outliers [4]. Modeling with artificial neural networks (ANN) overcomes many of these shortcomings by implementing many smaller models to interpret sections of data [5].

An ANN is an algorithm which finds relationships by performing discrete computations in artificial “neurons” and fitting these computations into a larger model [6]. Each neuron in an ANN models data using a distinct activation function [6–8]. ANN can be optimized by changing the number of neurons and type of activation functions [5, 7, 9]. Although previous bioprocess ANN approaches have shown success, they primarily rely on complex machine learning techniques and frequently require coding experience to implement [2, 8, 10–12].

Design of experiments (DOE) is a method of employing mathematics to generate optimal experimental conditions [13–15]. DOE serves two distinct roles in our project. The first is to establish experimental conditions which test four bioprocess inputs for their ability to maximize cell proliferation. We then model this bioprocess dataset using standard least squares (SLS) regression and ANN [16]. DOE’s second use in our project is to evaluate neuron activation functions and quantities in each layer of the ANN. By creating a DOE which optimizes an ANN using neuron numbers and activation functions as inputs and model outputs as desirability functions, we can systematically explore relationships to find the optimal ANN architecture [17, 18]. DOE in this context replaces traditional machine training activities with directed, mathematically optimized, exploration of inputs and outputs. Therefore, the ANN-DOE approach ensures appropriate and efficient leverage of ANN to maximize cell proliferation. Further background on the terms and techniques used in this manuscript can be found in Online Resource 1.

A well modeled bioprocess shows us which combination of inputs results in optimal outputs. To test the various modeling approaches, we utilize a bioprocess unit operation related to the manufacture of an allogeneic cell therapy for low back pain, which is currently in clinical evaluation (Clinicaltrials.gov NCT03347708 and NCT03955315) [19]. In this case study, we test how the cell seeding density, media supplement percentage, media exchange volume during routine feeding, and cell line maximize cell doublings.

In addition to modeling our bioprocess dataset with SLS regression and ANN, we test a historical bioprocess set point optimized with one factor at a time (OFAT) experimentation. Growing cells at each set point allows us to directly compare the relative merits of OFAT and DOE process optimization, as well as the value of modeling a bioprocess dataset with SLS regression or ANN-DOE hybrid modeling. We hypothesize that applying DOE to ANN architecture will result in an optimized network which we can use to model our bioprocess and improve cell doublings compared to a linear regression, OFAT-derived set point, or our historical process set point. If successful, this approach will improve the process with optimized set points and provide a valuable tool with which to assess future bioprocess operations.

## Materials and methods

All analyses and modeling were conducted using JMP Pro v. 14.0 on a 2019 MacBook Pro running Mojave 10.14.6.

### Establishing a DOE bioprocess dataset

Intervertebral disc material was obtained from two recently deceased donors under IRB approval and transported to the lab in Hypothermasol (Biolife Solutions) containing gentamycin (Mediatech) and amphotericin B (Mediatech). Each donor was processed independently into a distinct cell line. Nucleus pulposus tissue was dissected from the intervertebral discs using scalpels and tweezers. Cells were isolated from nucleus pulposus tissue using NB5 collagenase (Nordmark). Isolated cells were expanded in vented cap T150 attachment culture flasks (Corning) in the presence of DMEM/F12 (Corning), amphotericin B (Mediatech), gentamycin (Mediatech), and a cocktail of other proprietary media supplements. Temperature and pH were passively controlled by growing cells in a 37 °C, 5% CO_2_, incubator. At confluency, cells were dissociated from the flask using TrypLE (Thermo Fisher Scientific), formulated in 90% Characterized Fetal Bovine Serum (Hyclone), 10% DMSO (Protide Pharmaceuticals), and cryopreserved at − 196 °C for subsequent use.

At the time of testing, cells were thawed at 37 °C, washed with phosphate-buffed saline (pH 7.2; Thermo Fisher Scientific), and counted using a K2 automated cell counter (Nexcellom Bioscience). Cells were then passaged onto T25 attachment culture flasks (Corning) and grown under the same conditions as above except in the case of DOE parameters. Following 7 days of growth, cells were dissociated from the flask and counted using the K2 automated cell counter. Doublings were calculated using the formula: doublings = 3.32[log (total viable cells at harvest/total viable cells at seed)]. Microscopic images of the attached cells were obtained immediately prior to harvest.

Twenty-four unique conditions were derived from a D-optimal DOE. DOE input parameters were *cell line*, *seeding density*, *media supplement percentage,* and *media exchange percentage.* Each input parameter was investigated from the lowest (− 1) to highest (+ 1) points of our historically investigated ranges except for *cell line,* which accounts for two unique allogeneic cell lineages. All primary interactions, 2nd level interactions, and 2nd level powers were given necessary estimability in the DOE dialog with the response output as *doublings*. Finally, an SLS regression model of the bioprocess dataset was created.

### Analysis of bioprocess using DOE of neural network architecture.

In our ANN-DOE hybrid, 32 feedforward neural networks were constructed. Each ANN model in this exercise models the ability of *cell line*, *seeding density, media supplement percentage*, and *media exchange percentage* from the bioprocess dataset to predict *doublings*. The architecture of these 32 neural networks was chosen by running a D-optimal DOE with ANN node number and activation functions as input parameters (Table 1) and ANN output quality as response functions (Table 2). For ANN-DOE inputs, up to 100 linear, 100 TanH (sigmoid) and 100 Gaussian (bell curve) activation functions were evaluated at two levels each. ANN quality was evaluated using the coefficient of determination (*R*^2^) and Standard Square Error (SSE) for the training model as well as the difference between *R*^2^ and SSE between the training and validation datasets.

**Table 1:** Neural network DOE input levels

Input factors	− 1	0	1
First layer TanH	0	50	100
First layer Linear	0	50	100
First layer Gaussian	0	50	100
Second layer TanH	0	50	100
Second layer Linear	0	50	100
Second layer Gaussian	0	50	100

**Table 2:** Neural Network DOE response functions

Responses	Function
*R*^2^ Training	Maximize
SSE Training	Minimize
R^2^ Fit = *R*^2^ Training – *R*^2^ Validation	Minimize
SSE fit = SSE Training – SSE Validation	Minimize

All primary terms, 2nd level interaction terms, and 2nd level powers estimability were set to necessary. Five random starts were employed for each ANN. The bioprocess dataset was randomly split into a training (*n* = 16) and validation (*n* = 8) dataset. The training and validation datasets were maintained for all models. To find our optimal ANN architecture, we made an SLS regression model of the ANN-DOE. A new ANN of the bioprocess unit operation was created using the maximally desirable ANN architecture.

The doublings predicted by our training set versus the actual doublings observed in our validation set were compared for goodness of fit in the JMP Pro 14 Compare Model dialog. If successful, our optimized ANN should have a higher *R*^2^ and lower average absolute error (AAE) than a linear regression model or the non-optimized neural networks, when measuring the goodness fit for doublings.

### In vitro model qualification

Cells were grown in triplicate using the optimal *seeding density*, *media supplement percentage, cell line*, and *media exchange percentage* as defined by the following models: the SLS regression theorized optimum, the ANN theorized optimum, our OFAT set point, and a historical set point. Average doublings and standard deviation (SD) for each condition were calculated. A successful run will have a statistically improved doublings compared to our OFAT or historical set point, as measured by an LSMeans Differences Student’s *T* test at an α of 0.050.

## Results

### Model creation and evaluation

**Fig. 1:**
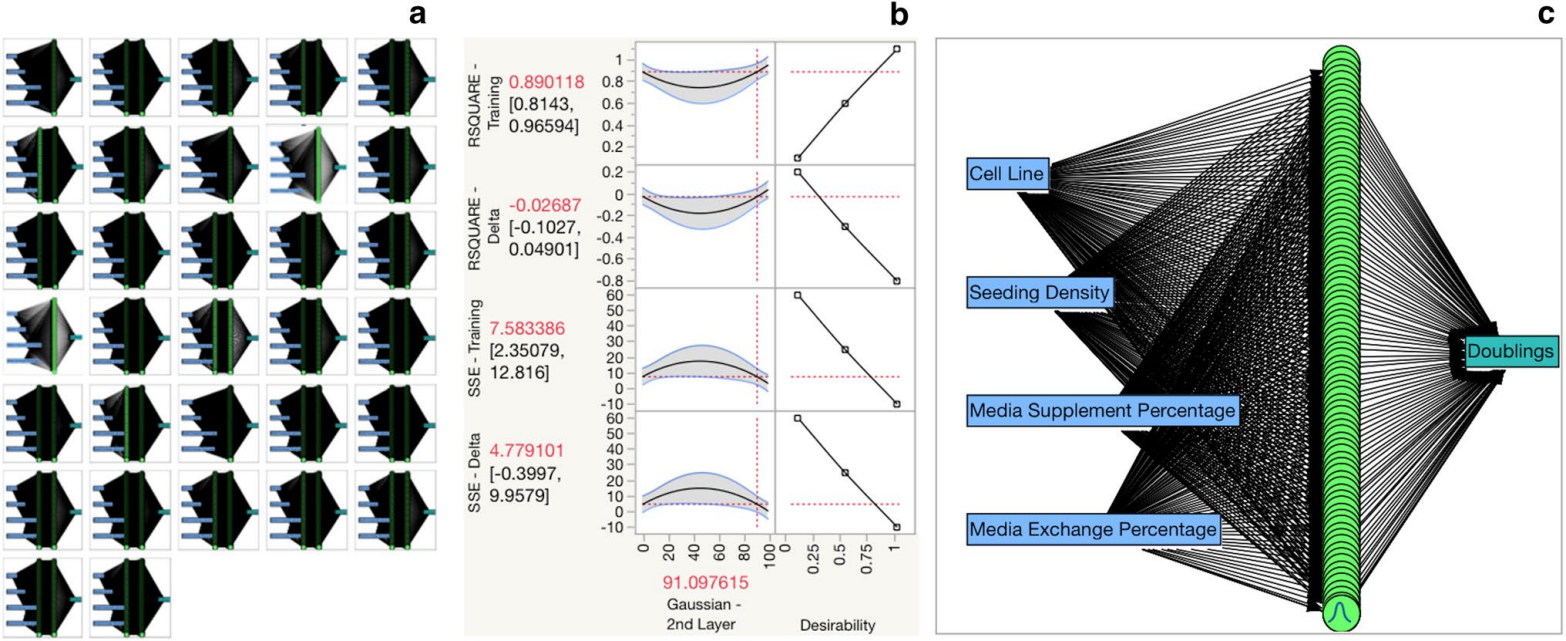
**a** Creating ANN-DOE Hybrid 32 ANNs are created per DOE specifications. **b**: ANN-DOE Hybrid Output parameters are modeled using standard least squares regression and the prediction profiler to find the optimal ANN configuration, a single layer ANN with 91-gaussian neurons. **c**: The Optimal ANN A new ANN is made using 91-gaussian activation functions Fig. 1 was created in JMP Pro 14

**Fig. 2:**
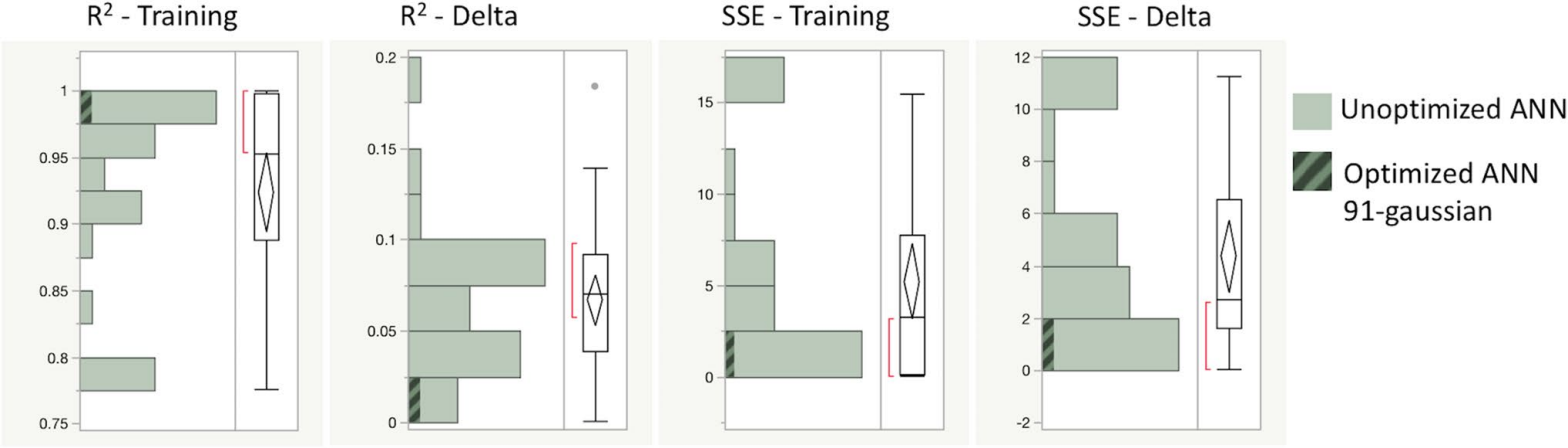
ANN Models Quality Distribution A quantile box plot of the ANNs is shown. The optimized ANN is highlighted with diagonal hatches. The goal was to maximize *R*^2^ Training and minimize the other outputs. We can see that our optimized ANN performs in the highest category for each quality metric. Figure 2 was created in JMP Pro 14. Data available in online resource 3

This bioprocess DOE was evaluated using 32 unoptimized ANN (Fig. 1a). The 32 unoptimized ANNs were modeled using SLS regression. Parameters with an effects test *p* > 0.05 were removed from the regression model. Desirability of R^2^ Training was maximized, whereas *R*^2^ Delta, SSE-Training, and SSE-Delta response values were minimized (Fig. 1b). By providing equal weight to all four model output functions, the maximum desirable ANN was determined to be a 1-layer, 91 Gaussian ANN (Fig. 1c). All ANN response values were recorded (Online Resource 2) and compared (Fig. 2) (Online Resource 3):

*R*^2^ Training represents our ANN ability to model the training dataset. Our DOE-ANN hybrid (*R*^2^ Training = 0.97) outperformed the mean unoptimized ANN (Mean *R*^2^ Training = 0.92, SD 0.08). The improvement suggests that our modeling approach improved our ability to model our training dataset.

The R^2^ Delta values represent the difference between *R*^2^ values in our training and validation data sets. The DOE-ANN model had the lowest *R*^2^ Delta value (*R*^2^ Delta 0.01) of all ANN tested (Mean *R*^2^ Delta 0.06, SD 0.4). Reduction in our *R*^2^ Delta value suggests that our ANN has an improved ability to model true process relationships, rather than overfitting or underfitting our data. This optimized *R*^2^ Delta value is more important than the *R*^2^ Training value because it is an indication of consistency between training and validation data sets.

SSE-Training represents the error in our training model calculated as the sum of squares for error. The DOE-ANN model (SSE-Training = 1.69) outperformed the mean (SSE-Training = 5.24, SD 5.70) of the unoptimized models. The improvement of SSE-Training in our optimized model suggests a reduced rate of error in our training dataset due to our DOE-ANN modeling approach.

SSE-Delta represents the difference between SSE values in our training and validation data sets. The optimized ANN-DOE (SSE-Delta = 0.83) outperformed the mean SSE-Delta from the 32 models (Mean SSE-Delta = 4.37, SD 3.82). The improvement in *R*^2^ Training alongside the simultaneous reduction in SSE-Training, R^2^ Delta, and SSE-Delta suggests that we improved the ANN modeling capacity while simultaneously reducing error compared to 32 unoptimized models with a wide range of model architectures.

**Fig. 3:**
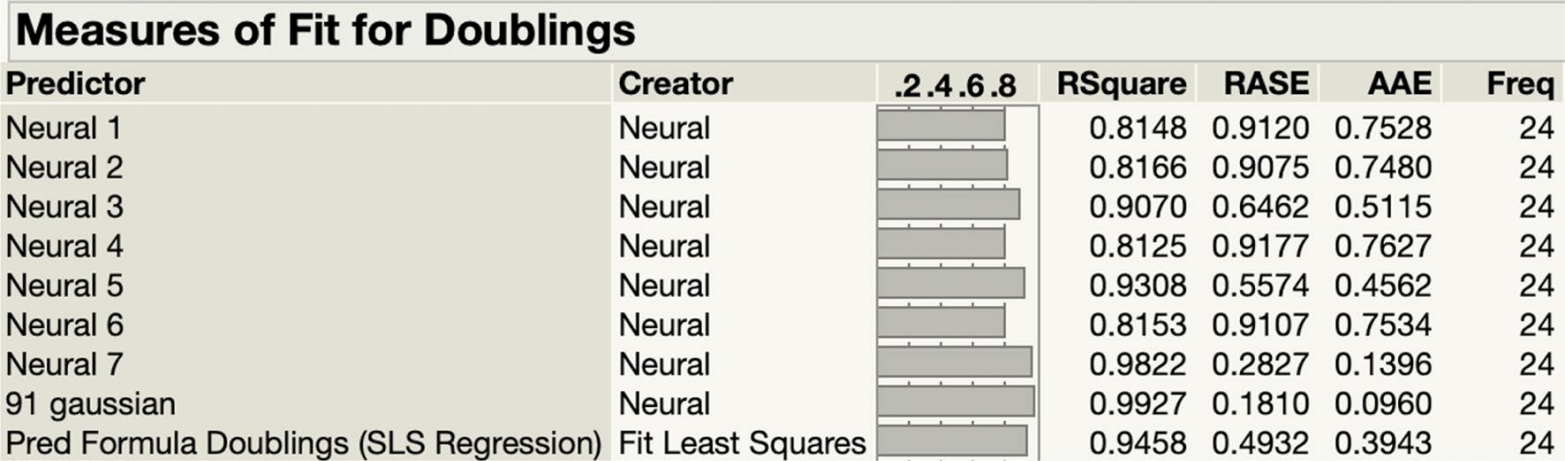
ANN versus Standard Least Squares Model Comparison. The models were compared using the model comparison dialog in JMP. This table shows the measure of fit of predicted doublings versus the actual doublings using the 24 runs from the historical dataset. Shown are the 7 neural nets with the highest Measure of Fit for Doublings *R*^2^ of the 32 created, the 91-Gaussian neural net, and the standard least squares model. Median of root average squared error (RASE) and average absolute error (AAE) for each model are also shown. Figure 3 was created in JMP Pro 14

In addition to direct comparison of the ANN-DOE response functions, models were also compared using the model comparison dialog in JMP Pro v 14.0 (Fig. 3). Specifically, the doublings predicted by our training set versus the actual doublings observed in our validation set were compared for goodness of fit using the 33 ANNs (32 from the ANN-DOE, plus one optimized ANN) and an SLS regression model of the bioprocess dataset. There are three key model comparability results. First, compared to the SLS regression model (*R*^2^ = 0.95, AAE 0.39), some of the 32 unoptimized ANNs created have lower *R*^2^ and higher error (*R*^2^ = 0.81, AAE = 0.76), whereas some have higher R^2^ and lower errors (*R*^2^ = 0.98, AAE = 0.13). This underperformance by some but not all of the 32 unoptimized ANNs shows that not every ANN is capable of improving SLS regression modeling capability. Second, the optimized ANN (*R*^2^ = 0.99, AAE of 0.10) had higher *R*^2^ and lower AAE than any of the 32 unoptimized models. This suggests that our ANN-DOE hybrid approach converged on a model with better modeling power and reduced error more than was possible by chance using 32 ANN with a wide range of architecture. Third, the optimized ANN outperformed the SLS regression model in both *R*^2^ (0.04 improvement) and AAE values (0.29 improvement). Together, these three key results show that not all ANNs outperform SLS regression and that our ANN is optimized to fit the validation dataset with more power and lower error than any of the other models generated, making it an optimal model for our bioprocess.

### In vitro model qualification

To test the real-world applicability of our in silico models, we tested the bioprocess set points in vitro. SLS regression and a 1-layer, 91 Gaussian ANN were each used to model the bioprocess DOE. When comparing the SLS regression optimized desirability set point versus the 91-Gaussian neural network optimized set point, the cell line and seeding density were calculated to be the same. However, the ANN determined that a larger percentage of the media supplement and a lower media exchange percentage could be beneficial when compared to the SLS regression optimum. The coded values for each condition are shown in Table 3 where −1 represents the lower end of the investigated range, 1 represents the high end of the investigated range, and 0 represents the center point.

**Table 3:** Process Setpoints and Theorized Optimum by Model

Models:	Cell Line	Seeding Density	Media Supplement Percentage	Media Exchange Percentage
SLS Regression Optimum	L1	−1	−0.462	−0.402
91-Gaussiun Optimum	L1	−1	0.358	−1
OFAT Setpoint	L1	−0.5	0	0
Historical Setpoint	L1	1	0	0

**Fig. 4:**
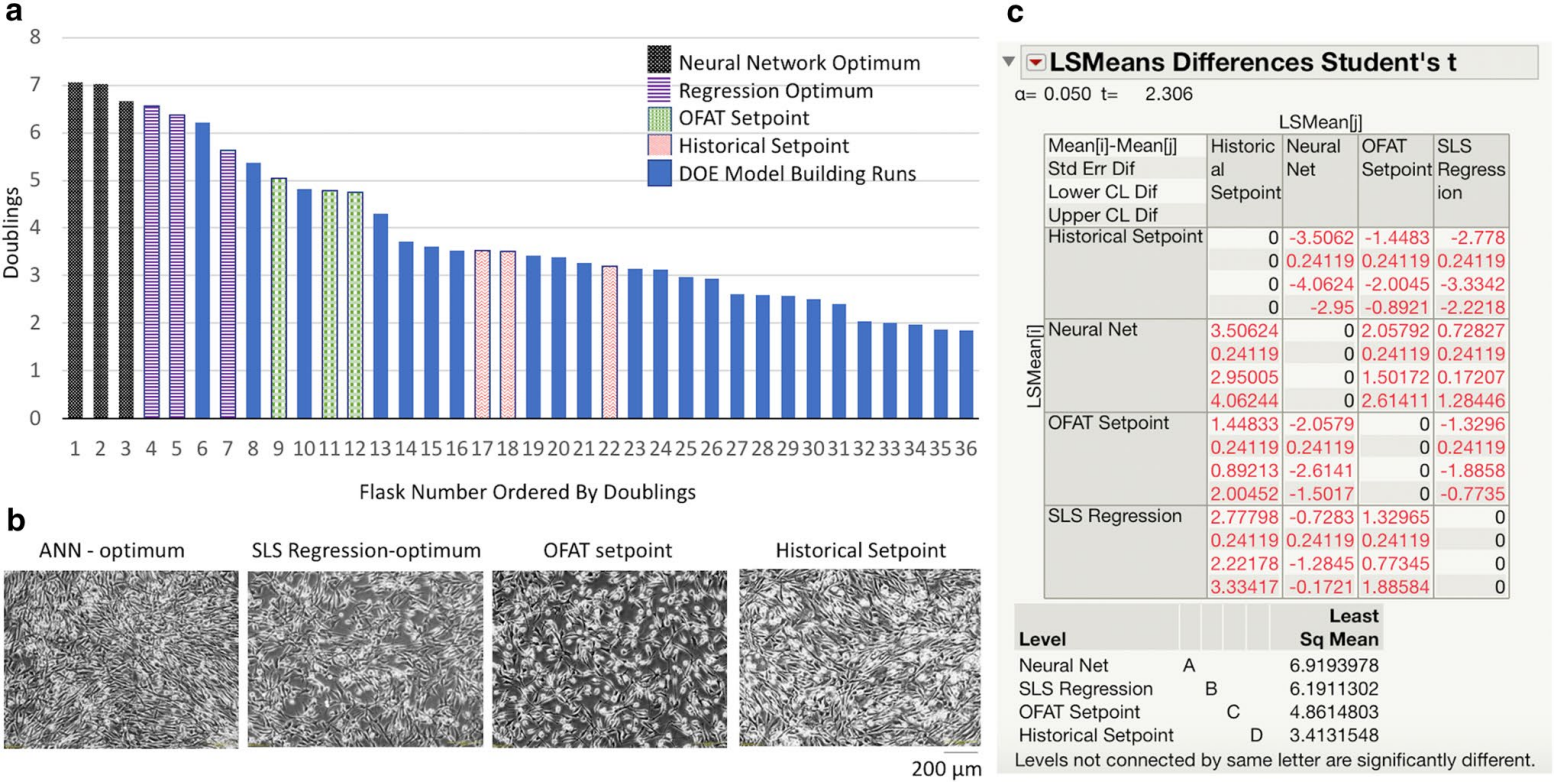
**a**: Doublings of all Flasks by Condition Doublings from the 24 DOE runs used to create the bioprocess design space and the 12 flasks grown for model comparison are graphed. The flasks grown under historical and OFAT conditions underperform many of the DOE runs. Two out of three replicates grown under regression derived setpoints outperform all cells grown under DOE conditions. All three replicates grown under ANN-derived conditions outperform all 33 other flasks. **b**: Cell Morphology by Condition Visually all cells exhibit standard “fibroblast-like” morphology. However, differences in cell density are observable between conditions. **c**: LSMeans Differences Student’s *T* Each condition is compared to every other condition with a *t* test at *a* = 0.050. We see that all four experimental setpoints resulted in significantly different doublings. Figure 4a, b were created in Microsoft Office Suite. Figure. 4c was created in JMP

Flasks were grown in triplicate using the bioprocess DOE set points, historical set point, OFAT set point, Regression Setpoint, and 91-Gaussian Setpoint. After harvesting all flasks, doublings were calculated (Fig. 4a). Visually all cells exhibit the expected elongated fibroblast-like morphology (Fig. 4b). Flasks grown using the historical set point and ANN are the most confluent. However, as predicted, the historical set point resulted in lower doublings.

The flasks grown using OFAT optimization performed significantly better (4.86 doublings, SD 0.15) than flasks grown at our historical set point (3.14 doublings, SD 0.18). However, many of the flasks grown during our DOE runs outperformed the OFAT optimization flasks. This outcome demonstrates that there are still significant gains to be made by looking at multiple variables in a DOE fashion.

The flasks grown using the SLS regression modeling (6.19 doublings, SD 0.49) significantly outperformed those optimized with OFAT experimentation. Further, of the three flasks grown using our SLS modeled set points, two outperformed all DOE runs. This performance shows that we found a true process relationship which allows us to significantly and reliably improve cell growth compared to our OFAT experimentation. However, the SLS model and ANN model did not agree on optimal set points.

All three flasks grown in ANN theorized optimum (6.91 doublings, SD 0.21) outperform all 33 other conditions. This demonstrates that our optimized ANN modeled our bioprocess better than our SLS regression model. The performance of the ANN suggests that a process optimum was found and modeled with fidelity which we were unable to be capture with a regression line. The ANN optimum also outperformed all DOE runs, some of which had more frequent media exchanges, and higher percentages of media supplement. Thus, using the ANN model allows for improved growth with reduction in media which will save on resources.

Finally, the 12 in vitro model qualification flasks were modeled with SLS regression and evaluated with post hoc tests. The regression model exhibited significance as measured by ANOVA (*p* < 0.01, *R*^2^ = 0.97) (Online Resource 4). Comparisons of least squares mean show that the ANN set point increased doublings by 0.69, 2.05, and 3.77 over the SLS setpoint, OFAT setpoint, and historical setpoint, respectively. Further, there were statistically significant differences between all four experimental groups as measured by Least Squares Means Differences Students Tukey at *α* = 0.05 (Fig. 4c). The statistically significant differences in doublings between all four groups show that the different models do grant different levels of process understanding, resulting in different abilities to optimize cell growth. The 11.6% improvement in cell doublings between the ANN- and SLS-derived setpoints suggests that a process optimum was found and modeled with fidelity which we were unable to be capture with a regression line. The ANN also outperformed all DOE runs, some of which had more frequent media exchanges, and higher percentages of media supplement. Thus, using the ANN model allowed for improved growth with reduction in media which will save on resources.

## Discussion

Improvement of process understanding without the need for laboratory experiments is inherently attractive to the bioprocess professional. In silico, our optimized ANN performed well for our quality outputs *R*^2^ Training, SSE Training, *R*^2^ Fit and SSE Fit. These high-quality outputs indicate that our DOE approach found an improved ANN configuration compared to 32 unoptimized ANN. Further, our ANN demonstrated better fit for doublings with lower error than all 32 non-optimized ANN and the SLS model. This improvement in model fit suggests that our ANN-DOE approach models true process relationships that were not previously captured with SLS regression or unoptimized ANN models.

In vitro, we exhibited differences between each method of bioprocess development. The ANN-DOE showed 11.2% improvement in doublings over SLS regression, and 42.2% improvement over OFAT experimentation. The comparison of development pathways shows how a DOE dataset can significantly improve a process compared to OFAT experimentation regardless of whether ANN or SLS regression is used to model the data set. However, using the ANN-DOE, we found a process set point which significantly improved bioprocess outcomes compared to the already capable linear regression model.

This manuscript describes four tiers of bioprocess development efficiency, tests them rigorously with in silico and in vivo methodologies, and demonstrates statistically significant differences between each process outcome. Between the improved modeling capability in silico and significant increase in process doublings *in vitro*, we can conclude that our ANN-DOE hybrid approach to process development efficiently leveraged ANN to improve bioprocess outputs beyond the capabilities of an SLS regression model. The improvement of 0.69 doublings over SLS regression, and improvement of 2.05 doublings over OFAT experimentation indicates that the approach should be tested on other bioprocess operations.

The value of the ANN-DOE approach, beyond achieving an improved process model, is that it bypasses the complex training and validation exercises generally used in machine learning in favor of evaluating at the network architecture using DOE techniques already familiar to many bioprocess professionals. Modeling of an established bioprocess dataset in this manner is relatively quick and inexpensive to conduct.

Using DOE to evaluate machine learning models, a single scientist can use a laptop to improve the process set points using only a few hours of work and somewhere between a day to a week of computing power, depending on the complexity of the model and power of the computer. It is likely that the greatest value of ANN modeling will come not from evaluation of development studies but from modeling perturbations of established bioprocess manufacturing operations. However, given that ANNs are notoriously unreliable [5], we recommend always verifying manufacturing set points using a small-scale model before making drastic changes in the manufacturing process.

Despite its preliminary success, the combination of DOE with ANN could be improved upon in several ways. Increasing the size of the bioprocess dataset or augmenting our ANN-DOE hybrid model using standard DOE augmentation techniques would result in higher fidelity models. Only 3 neuron types are discussed here, but the DOE principals herein should be applicable for any neuron type and for many deep learning problems. Although either a traditionally boosted ANN or a DOE derived ANN is sufficient to model a process, further investigation into the harmonization of the two approaches would be beneficial.

With the immense value derived from process improvements for pharmaceuticals, and the rapid turnaround time of the ANN-DOE Hybrid approach, ANNs can provide an incredible investment to reward ratio. Beyond its application in the bioprocess field, we believe the principals herein may offer a robust and thoughtful way to explore the creation of ANN architecture across disciplines.

## Supplementary Information

Below is the link to the electronic supplementary material.

Supplementary file1 (PDF 560 KB)

Supplementary file2 (PDF 148 KB)

Supplementary file3 (PDF 118 KB)

Supplementary file4 (PDF 206 KB)

## Data Availability

Additional Data in Supplemental Materials.
