# Supplementary material for: Design of experiment (DOE) applied to artificial neural network architecture enables rapid bioprocess improvement
Source: Bioprocess Biosyst Eng. 2021 Feb 27;44(6):1301–8. doi: 10.1007/s00449-021-02529-3 (PMC8144078; doi:10.1007/s00449-021-02529-3)
Supplement: Supplementary file 1 — Supplementary file1 (PDF 560 KB) [file 449_2021_2529_MOESM1_ESM.pdf]

Online Resource 1 for Design of Experiment (DOE) Applied to Artificial Neural Network Architecture Enables Rapid Bioprocess Improvement

Journal: Bioprocess and Biosystems Engineering

Daniel Rodriguez-Granrose[1,2], Amanda Jones[1], Hannah Loftus[1], Terry Tandeski[1], Will Heaton[1], Kevin T Foley[1,3,4], Lara Silverman[1,3]

Affiliations:

[1] DiscGenics Inc, Salt Lake City, Utah, USA
[2] Department of Biochemistry and Molecular Biology, University of Miami, Miami, FL, USA
[3] Department of Neurosurgery, University of Tennessee Health Science Center, Memphis, Tennessee, USA
[4] Semmes-Murphey Clinic, Memphis, Tennessee, USA

Corresponding Author:
Daniel Rodriguez-Granrose
daniel@discgenics.com

Background of Artificial Neural Networks and Design of Experiments

This online is intended to provide the reader with the background and definitions sufficient to understand Artificial Neural Networks (ANN) and Design of Experiments (DOE) at the level required to implement our ANN-DOE approach.

Artificial Neural Network Architecture:

An ANN is an algorithm which finds relationships by clustering raw data into recognized patterns. [6] ANNs perform discrete computations in artificial "neurons." [6] Each neuron combines input data with weights that either amplify or dampen input based on how well they can describe data without error. [5] The neurons which can describe a portion of the process, are "activated" and work together to model the data. [5] Conversely, those which do not accurately describe data are not "activated" and do not provide any input into subsequent layers of an ANN.

Each neuron in an ANN models data using a distinct activation function. [5] Analysis of activation functions has shown that specific problems call for a specific set of activation functions. [7] While there are dozens of types of activation functions, we will use three common activation functions: linear, TanH (s-shaped), and Gaussian (bell curve shaped).

ANN Training and Validation:

An ANN uses a training data set to create the model and a validation data set to qualify performance. At first use, an ANN employs unoptimized weights and activation functions, and

consequently does a poor job at modeling a data set. [5] Training a model with successive iterations can improve model outcomes. [5] However, training an ANN is one of the most difficult and time-consuming parts of the process. [2] To improve upon the initial analysis, many sophisticated neural networks employ a combination of model boosting techniques, learning rates, random seeds, model tours, and cross-validation. [9]

A *feedforward neural network* is a type of ANN in which neurons stack several layers deep and the output from each layer feeds into subsequent layers. [7] A feedforward model is dubbed feedforward because each layer informs layers deeper in the model, and the movement of data only goes forward, never backwards. [5] An example of this type of network is shown in **Online Resource 1 (OR1) Figure 1**, which depicts a bioprocess that includes factors such as the seeding density of the cells, the percent of a given media supplement, the percent of media exchanged during routine feeding, and what cell line is used. Ultimately, these factors can work together to determine the final number of cell doublings, which is a typical key process attribute in such bioprocess operations.

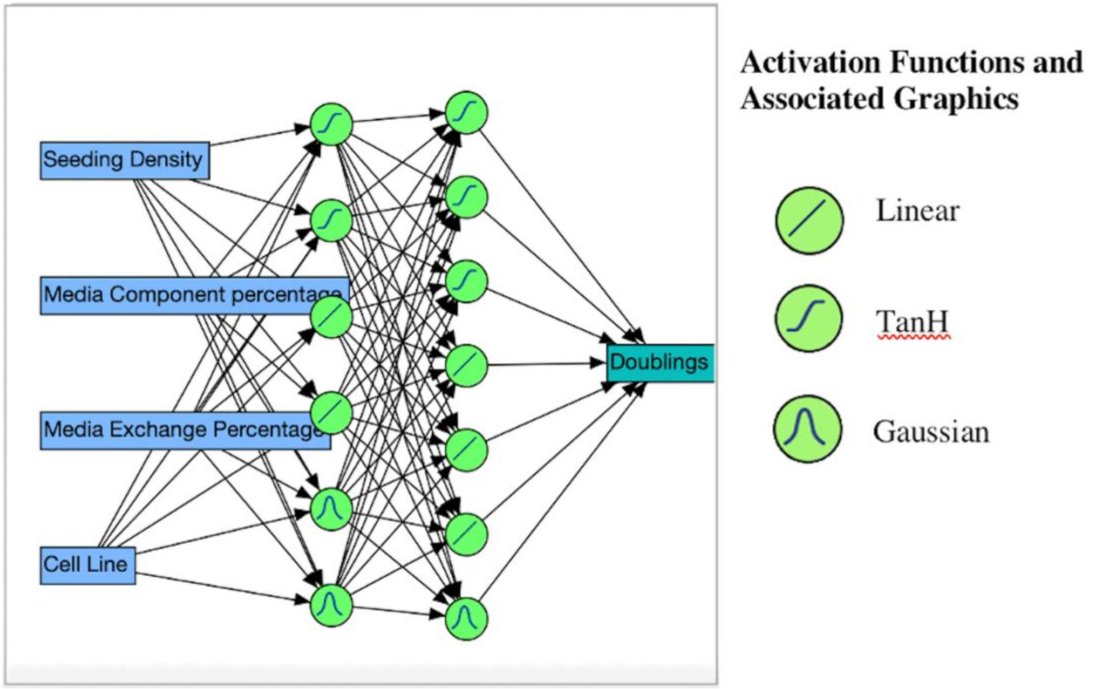

OR1 Fig 1 Feedforward Neural Network Example of a feedforward neural network in which two layers of neurons model the effect of seeding density, percentage of a media supplement, media exchange percentage, and cell line on doublings. Here the first layer of neurons models raw input data, a second layer models the relationships between the first layer of neurons and process doublings. The graphic inside each neuron serves as a representation of which activation function is used to model data

- A *linear activation function* multiplies inputs by the weights generated for each node and creates an output signal proportional to that input. [5] A node with linear activation

functions as a linear regression model. [5] It has limited flexibility in handling complex datasets. [5]

- A *TanH activation function* is an s-shaped function that outputs. [7] The benefit of a TanH function is that it is nonlinear, allowing it to map more complicated functions, stack layers, and obtain high accuracy. [7]

- A *Gaussian activation function* is a continuous bell curve shaped function which interprets output depending on how close the input is to the average. [7]

Together, these techniques enable the ANN to "learn" how to model the data with increased accuracy. However, when the data modeled does not reflect a true population, these techniques can sometimes overfit or overfit data rather than model true relationships. [5] An overfit model predicts the fitted data very well but predicts future observations poorly. To mitigate overfitting, one option is to use an independent data set to assess the predictive power of the model. As a default in JMP, one third of data points are modeled in an independent "cross-validation" model. [9,5] An ANN which neither overfits nor underfits the data will have similar coefficient of determination (R^2) and Standard Square Error (SSE) in the training and validation data set.

<u>ANN Training and Validation:</u>

An ANN uses a training data set to create the model and a validation data set to qualify performance. At first use, an ANN employs unoptimized weights and activation functions, and consequently does a poor job at modeling a data set. [5] Training a model with successive iterations can improve model outcomes. [5] However, training an ANN is one of the most difficult and time-consuming parts of the process. [2] To improve upon the initial analysis, many sophisticated neural networks employ a combination of model boosting techniques, learning rates, random seeds, and model tours. Some approaches include the following: [9]

- *Boosting* is a general method for improving the performance of a learning algorithm. It works by creating an additive sequence of ANN models, scaled by the learning rate, into a larger model.

- *Learning rates* control how much to change the model in response to the estimated error each time the activation function weights are updated. The learning rate must be $0 < r \leq 1$. Learning rates close to 1 result in faster convergence on an ultimate model, but also have a higher tendency to overfit data.

- *Random seeds* are used in ANN to randomize initial activation function weights.

- *Tours* are the number of times an ANN is run from a blank state. Because initial weights are randomized, having multiple tours can result in higher quality ANNs.

Together, these techniques enable the ANN to "learn" how to model the data with increased accuracy. However, when employed aggressively, these techniques tend to overfit data. [5] An overfit model predicts the fitted data very well but predicts future observations poorly. To

mitigate overfitting, it is important to use an independent data set to assess the predictive power of the model. Typically, one half to one third of data points should be modeled in an independent "validation" model. [9,5] An ANN which neither overfits nor underfits the data will have similar R^2 and SSE in the training and validation data set.

When the estimate of an output includes the influence of one or more inputs the effects are said to be correlated. [4] A multivariate experiment with too few experimental conditions is highly correlated, and thus unable to distinguish the source of unique signals. A multivariate experiment where each input is individually tested has low correlation but can be prohibitively expensive.

DOE is a method of employing mathematics to generate optimal experimental conditions. By minimizing the correlation coefficient, custom DOE provides a maximum amount of statistically significant information from a given number of experimental conditions. [15,13] By changing multiple factors at a time, DOE helps develop a multi-dimensional dataset. Subsequent analysis of this dataset can reveal how changes in factors affect the overall model in the presence of many other factors. [13]

To perform DOE, it is necessary to define factors, responses, and model effects. Together these components allow the construction of a basic custom DOE.

- *Factors* are model inputs. In a bioprocess, factors might include pH and temperature. Each factor should have a center point identified as well as an upper and lower setpoint that is an equal distance above and below the center point. These points are often coded as -1, 0 and 1, with -1 and 1 representing the lower and higher ends of the investigated range respectively. *[14]*

- *Responses* are model outputs. In a bioprocess, responses might include population doublings or gene expression. For each response, a goal (maximize, minimize, or match) should be provided as well as an upper and lower limit of failure, where limits exist. [14]

- *Model effects* define which combination of *factor* effects can be estimated by the DOE. [14] Estimability can be set to *necessary* if the model effect needs to be modeled or *if possible* the model effects would be good but not necessary to know.

    - *Main effects* indicate the primary factor effects on responses. [14] For example, whether pH impacts population doubling.

    - *Interaction effects* indicate how various factors interact to effect responses. [14] For example, whether pH and temperature work together to predict doublings.

    - *Powers* indicate which level of polynomial function you can detect in a model. [14] When data cannot be accurately represented by a linear model, higher order functions can be used to fit data. For example, second power functions, also called quadradic functions, can model parabolic curvature in a dataset. Third power functions, also called cubic functions, can be used to model s-shaped datasets. It is common to only search for first or second order terms when constructing a DOE.

A critical aspect of the DOE is the use of coded levels. Coded levels allow for a direct comparison of different parameters by means of scale invariance. Giving each parameter a value at a center point and two to four points spaced equidistant from the center point allows us to look at our optimal operating range, curvature, and edges of failure. For example, assigning numbers for pH and temperature at values a, -1, 0, 1, and A allows for scale invariant statistical comparison even though they are measured with different units. “a” and “A” are sometimes included when modeling higher power terms. An example of setting coded levels for a bioprocess in a 2-dimensional space is shown in **OR1 Figure 2 a**. The values associated with each level are shown in **OR1 Figure 2 b** and coded variables by experimental condition are shown in **OR1 Figure 2 c**.

a

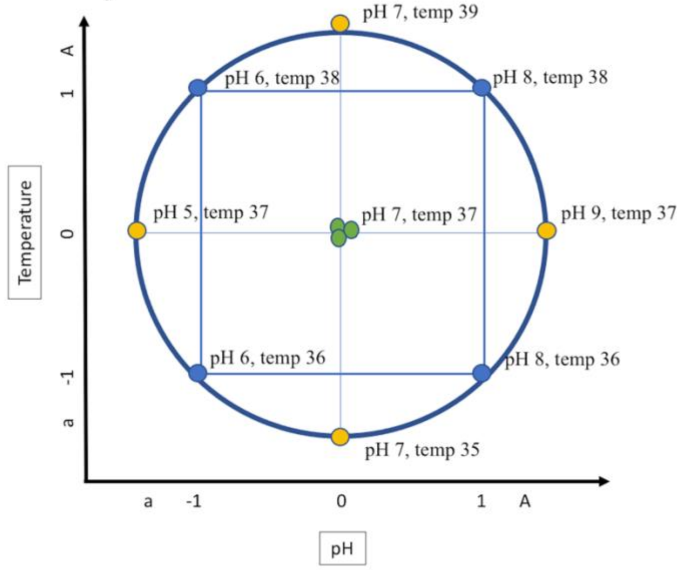

b

	a	-1	0	1	A
**pH**	5	6	7	8	9
**Temperature (C)**	35	36	37	38	39

c

Central Composite Response Surface DOE - 2 Factors, 2 Levels			
Condition	Pattern	Variable 1	Variable 2
1	00	0	0
2	00	0	0
3	00	0	0
4	--	-1	-1
5	-+	-1	1
6	+-	1	-1
7	++	1	1
8	A0	-1	0
9	A0	1	0
10	0a	0	-1
11	0A	0	1

OR1 Fig 2 a: Visual Representation of DOE This response surface model builds a knowledge space around pH and temperature using three center points to derive error, four corner points to assess interaction effects, and four star points to evaluate model curvature and expand the design space. **b: Table of Values at Each Level of the DOE** This table shows which values are used at a coded position in the example DOE **c: Table of DOE setpoints using coded variables** The table shows how coded variables represent specific combinations of conditions

An executed DOE results in a dataset with any number of inputs and outputs. As such, the types of analysis which can be performed on DOE are numerous. The two primary types of analysis we perform in the creation of our DOE here are feedforward ANN modeling and standard least squares (SLS) regression.

- *Least Squares Regression* is a type of linear regression commonly used to model bioprocesses. [3,1] The least squares method fits a line of process inputs to outputs by mapping process data points and plotting a regression line where the squared vertical distance from data points to the regression line is as small as possible. [16] This

regression analysis can then be used to interpolate the optimal process setpoint by finding the point in the line where inputs result in maximum output desirability. [16]

Due to overlapping terminology, we differentiate between sources of data and types of modeling used in this paper in **SM Table 1**. Historical bioprocess runs, bioprocess DOE experiments, and OFAT experiments are traditional sources of data. The output of our ANN DOE (R^2, SSE) is also treated as a source of data; whereas regression models and ANN are both ways of modeling the data. Finally the order of operations for the manuscript is outlined in **OR1 Figure 3.**

SM Table 1 Sources of Data vs Types of Modeling

**Sources of Data**	**Types of Modeling**	**Term used to Describe Optimum in This Paper**
Historical Bioprocess Experiments	None	Historical Bioprocess
Historical Bioprocess Experiments, augmented with OFAT experiments	Analysis of Variance (ANOVA)	OFAT Bioprocess
DOE of Bioprocess	SLS Regression	SLS Bioprocess
DOE of Bioprocess	ANN Architecture Optimum	ANN Bioprocess
DOE-ANN Hybrid Output	SLS Regression	ANN Architecture

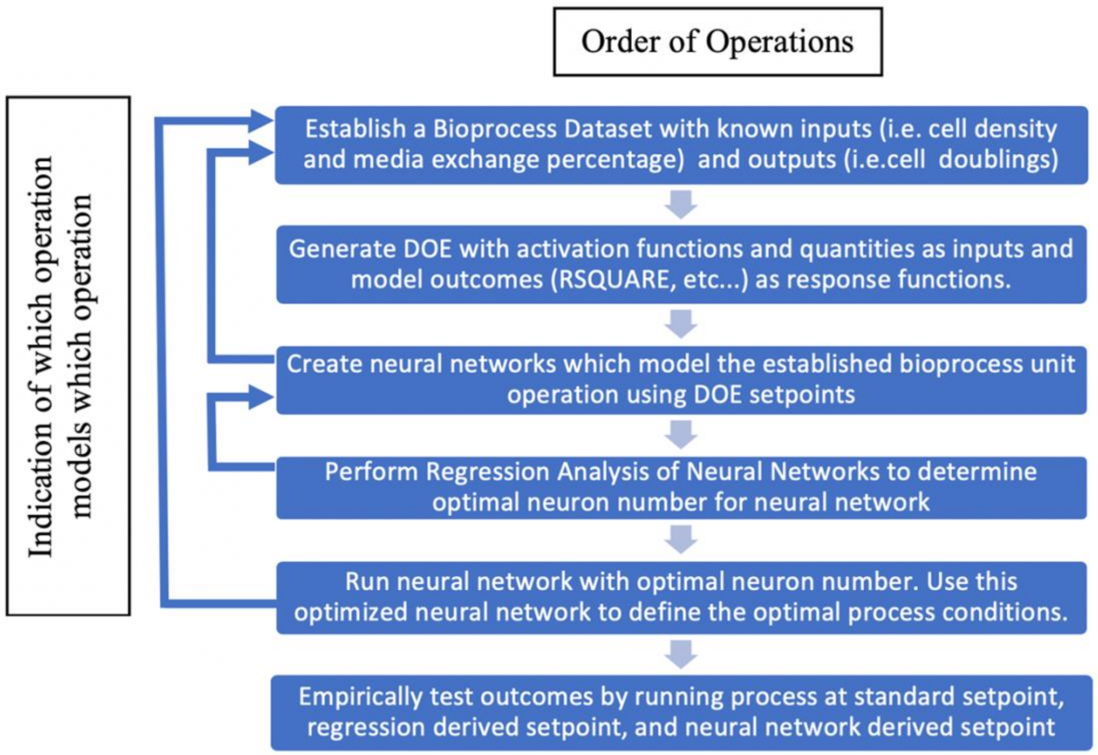

OR1 Fig 3 Flow Diagram for Creating and Testing Neural Network Bioprocess Model using DOE on Network Architecture The flow diagram shows the order in which the ANN is made from establishing a bioprocess dataset to empirical test of outcomes. The arrows on the left side of the flow diagram show what data is being modeled in each step. For example, the neural network in step 4 is modeling the process data from step 1. This graphic created in MS Word

References: shared with manuscript
