# Supplementary material for: Design of experiment (DOE) applied to artificial neural network architecture enables rapid bioprocess improvement
Source: Bioprocess Biosyst Eng. 2021 Feb 27;44(6):1301–8. doi: 10.1007/s00449-021-02529-3 (PMC8144078; doi:10.1007/s00449-021-02529-3)
Supplement: Supplementary file 2 — Supplementary file2 (PDF 148 KB) [file 449_2021_2529_MOESM2_ESM.pdf]

Online Resource 2 for Design of Experiment (DOE) Applied to Artificial Neural Network Architecture Enables Rapid Bioprocess Improvement

Journal: Bioprocess and Biosystems Engineering

Daniel Rodriguez-Granrose[1,2], Amanda Jones[1], Hannah Loftus[1], Terry Tandeski[1], Will Heaton[1], Kevin T Foley[1,3,4], Lara Silverman[1,3]

Affiliations:

[1] DiscGenics Inc, Salt Lake City, Utah, USA
[2] Department of Biochemistry and Molecular Biology, University of Miami, Miami, FL, USA
[3] Department of Neurosurgery, University of Tennessee Health Science Center, Memphis, Tennessee, USA
[4] Semmes-Murphey Clinic, Memphis, Tennessee, USA

Corresponding Author:
Daniel Rodriguez-Granrose
daniel@discgenics.com

	Inputs (ANN architecture used to model bioprocess dataset)					Outputs (How well the ANN models the bioprocess dataset)			
**TanH - 1st Layer**	**Linear - 1st Layer**	**Gaussian - 1st Layer**	**TanH - 2nd Layer**	**Linear - 2nd Layer**	**Gaussian - 2nd Layer**	**RSQUARE - Training**	**RSQUARE - Delta**	**SSE - Training**	**SSE - Delta**
0	0	100	0	0	0	0.999687	0.0711758	0.0215997	-2.3933148
100	100	0	100	0	50	0.1873044	0.7502407	56.087049	53.9773016
0	0	100	100	0	100	0.9993744	0.0762362	0.0431736	-2.5532447
100	0	100	0	100	0	0.9985026	0.1394487	0.1033434	-4.6578616
0	0	100	100	100	0	0.9997088	0.0695394	0.0200966	-2.3388036
50	100	50	50	0	0	0.9189367	0.0193819	5.5944703	3.5108509
0	0	100	0	100	100	0.999608	0.0743947	0.0270524	-2.499267
0	0	0	100	100	100	0.9189352	0.0371155	5.594576	4.1099548
0	0	0	100	0	0	0.9168164	0.0498224	5.7408028	4.6138508
50	50	0	100	0	100	0.9994455	0.0776931	0.0382676	-2.6049618
100	50	100	50	50	50	0.9005349	0.0438728	6.8644468	4.9865209
0	100	100	100	100	100	0.8358272	0.1015769	11.330157	9.2156471
100	100	100	0	0	100	0.9961297	0.0866709	0.2671026	-2.7914097
0	100	0	100	100	0	0.775615	-0.0922156	15.485614	11.0208884
0	50	50	50	0	100	0.9691776	0.0811206	2.1271661	-1.6543049
0	0	0	0	100	0	0.8836801	-0.0846751	8.0276525	6.958681
100	100	50	100	50	100	0.9740413	0.1837166	1.7915025	-5.2913975
50	0	0	0	0	50	0.9522421	0.0482541	3.2959418	0.0526238
100	0	0	100	100	0	0.7756116	-0.0938328	15.485848	11.0756368
100	100	100	100	100	0	0.7754898	-0.0984621	15.494256	11.2363088
100	50	0	0	100	100	0.9996096	0.0577091	0.0269458	-1.9356742
100	100	0	0	50	0	0.7756353	-0.092383	15.484214	11.0258289
100	50	50	0	0	0	0.9539576	0.0496907	3.1775544	-0.0563417
100	100	50	0	100	50	0.9441617	0.0369218	3.8536011	0.7201343
100	0	100	100	100	100	0.9566807	-0.0025323	2.9896205	1.6118228
50	0	50	0	50	100	0.9460516	0.0293787	3.7231761	0.9083586
50	100	0	50	100	100	0.7755625	-0.0910965	15.489241	10.9849368
0	100	100	0	100	0	0.7756256	-0.0934072	15.484884	11.0607693
100	0	100	100	0	0	0.9157224	-0.0342583	5.8163032	4.1266358
0	100	0	0	0	100	0.9997102	0.0581763	0.02	-1.955005
100	0	0	50	0	100	0.9653943	0.0498319	2.3882653	-0.4640645

**0**	**100**	**100**	**100**	**0**	**0**	**0.9983386**	**0.0213536**	**0.1146583**	**-0.6627966**

Online Resource 2 Table of ANN-DOE Hybrid Functions and Outputs The table shows architecture and responses of each ANN which modeled doublings using the bioprocess dataset. We used all values in this table, ran an SLS regression model of inputs functions on responses, removed model inputs which were not statistically significant. Using the maximize desirability function in JMP Pro 14 we found 91-94 gaussian neurons as the maximally desirable ANN configuration.
