# Supplementary material for: Design of experiment (DOE) applied to artificial neural network architecture enables rapid bioprocess improvement
Source: Bioprocess Biosyst Eng. 2021 Feb 27;44(6):1301–8. doi: 10.1007/s00449-021-02529-3 (PMC8144078; doi:10.1007/s00449-021-02529-3)
Supplement: Supplementary file 3 — Supplementary file3 (PDF 118 KB) [file 449_2021_2529_MOESM3_ESM.pdf]

Online Resource 3 for Design of Experiment (DOE) Applied to Artificial Neural Network Architecture Enables Rapid Bioprocess Improvement

Journal: Bioprocess and Biosystems Engineering

Daniel Rodriguez-Granrose[1,2], Amanda Jones[1], Hannah Loftus[1], Terry Tandeski[1], Will Heaton[1], Kevin T Foley[1,3,4], Lara Silverman[1,3]

Affiliations:

[1] DiscGenics Inc, Salt Lake City, Utah, USA
[2] Department of Biochemistry and Molecular Biology, University of Miami, Miami, FL, USA
[3] Department of Neurosurgery, University of Tennessee Health Science Center, Memphis, Tennessee, USA
[4] Semmes-Murphey Clinic, Memphis, Tennessee, USA

Corresponding Author:
Daniel Rodriguez-Granrose
daniel@discgenics.com

	Summary Statistics	R^2 Training	R^2 Delta	SSE Training	SSE Delta
	Mean	0.92	0.07	5.24	4.37
	Standard Deviation	0.08	0.04	5.70	3.82
	Standard Error Mean	0.01	0.01	1.01	0.68
	Upper 95% Mean	0.95	0.08	7.29	5.75
	Lower 95% Mean	0.89	0.05	3.18	2.99
	N	32.00	32.00	32.00	32.00
	Quantiles	R^2 Training	R^2 Delta	SSE Training	SSE Delta
maximum	100.00%	1.00	0.18	15.49	11.24
	99.50%	1.00	0.18	15.49	11.24
	97.50%	1.00	0.18	15.49	11.24
	90.00%	1.00	0.10	15.49	11.05
quartile	75.00%	1.00	0.09	7.74	6.54
median	50.00%	0.95	0.07	3.24	2.70
quartile	25.00%	0.89	0.04	0.11	1.62
	10.00%	0.78	0.02	0.02	0.52
	2.50%	0.78	0.00	0.02	0.05
	0.50%	0.78	0.00	0.02	0.05
minimum	0.00%	0.78	0.00	0.02	0.05

Online Resource 3 Table of ANN Quantile Summary Statistics The number here shows the values which complement quantile box plots within manuscript Fig 2: ANN Models Quality Distribution. The numbers from online Resource 3 were created using JMP Pro v. 14
