# Supplementary material for: Design of experiment (DOE) applied to artificial neural network architecture enables rapid bioprocess improvement
Source: Bioprocess Biosyst Eng. 2021 Feb 27;44(6):1301–8. doi: 10.1007/s00449-021-02529-3 (PMC8144078; doi:10.1007/s00449-021-02529-3)
Supplement: Supplementary file 4 — Supplementary file4 (PDF 206 KB) [file 449_2021_2529_MOESM4_ESM.pdf]

Online Resource 4 for Design of Experiment (DOE) Applied to Artificial Neural Network Architecture Enables Rapid Bioprocess Improvement

Journal: Bioprocess and Biosystems Engineering

Daniel Rodriguez-Granrose[1,2], Amanda Jones[1], Hannah Loftus[1], Terry Tandeski[1], Will Heaton[1], Kevin T Foley[1,3,4], Lara Silverman[1,3]

Affiliations:

[1] DiscGenics Inc, Salt Lake City, Utah, USA
[2] Department of Biochemistry and Molecular Biology, University of Miami, Miami, FL, USA
[3] Department of Neurosurgery, University of Tennessee Health Science Center, Memphis, Tennessee, USA
[4] Semmes-Murphey Clinic, Memphis, Tennessee, USA

Corresponding Author:
Daniel Rodriguez-Granrose
daniel@discgenics.com

a

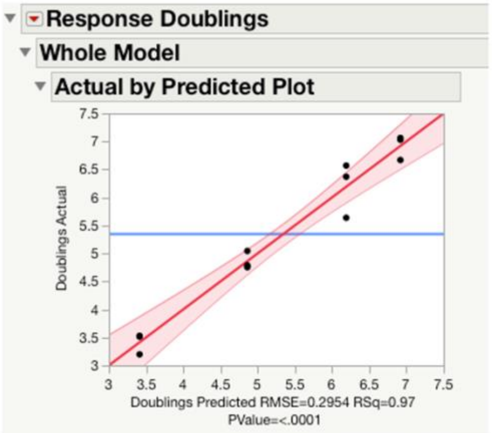

b

Summary of Fit

RSquare	0.968525
RSquare Adj	0.956722
Root Mean Square Error	0.295401
Mean of Response	5.346291
Observations (or Sum Wgts)	12

c

Analysis of Variance

Source	DF	Sum of Squares	Mean Square	F Ratio
Model	3	21.481425	7.16048	82.0572
Error	8	0.698096	0.08726	Prob > F
C. Total	11	22.179522		<.0001*

d

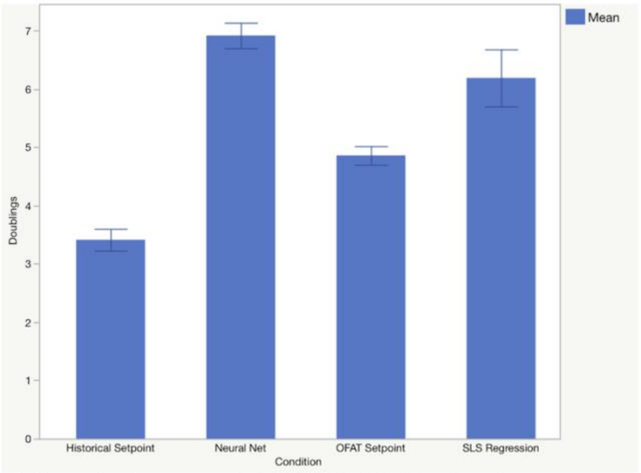

Each error bar is constructed using 1 standard deviation from the mean.

e

Condition	Doublings
SLS Regression	6.369. 6.586, 5.635
OFAT	4.753, 5.043, 4.787
Historical Setpoint	3.197, 3.513, 3.528
Neural Net	7.062, 6.668, 7.029

Online Resource 4 a: Regression Model of the Four Tested Bioprocess Setpoints The regression model compares doublings compared by our four process setpoints. We show a strong regression line with **b: summary of fit** (R^2 = 0.97), **c: ANOVA table** (P < 0.001) and actual values. This model provides the basis for Fig 4C within the manuscript **d: doubling bar graphs**

and **e: doubling values for individual flasks by bioprocess setpoints** Online Resource 4 was created using JMP Pro v. 14
